# Malnutrition Is Associated with Increased Liver Stiffness in Type 2 Diabetes: The Mediating Role of Inflammation

**DOI:** 10.3390/biom15121735

**Published:** 2025-12-13

**Authors:** Aurelio Lo Buglio, Francesco Bellanti, Rosanna Villani, Cristiano Capurso, Grazia Pia Magnati, Sara Cioffi, Gabriele Tedesco, Carlo Alberto Torsello, Gianluigi Vendemiale, Gaetano Serviddio

**Affiliations:** Department of Medical and Surgical Sciences, University of Foggia, Viale Pinto 1, 71122 Foggia, Italy; francesco.bellanti@unifg.it (F.B.); rosanna.villani@unifg.it (R.V.); cristiano.capurso@unifg.it (C.C.); grazia.magnati@gmail.com (G.P.M.); sara_cioffi@icloud.com (S.C.); gabtedesco95@gmail.com (G.T.); torcarlo94@gmail.com (C.A.T.); gaetano.serviddio@unifg.it (G.S.)

**Keywords:** liver fibrosis, malnutrition, inflammation, MASLD, chronic low-grade inflammation

## Abstract

Background: Malnutrition is a prevalent and under-recognized condition in patients with type 2 diabetes mellitus (T2DM), contributing to various complications, including liver fibrosis. In this study, we aimed to evaluate the association between malnutrition and liver fibrosis in patients with T2DM, and to assess whether inflammation mediates this relationship. Methods: In this prospective single-centre study, 87 adult outpatients with T2DM underwent nutritional assessment using the Subjective Global Assessment (SGA) and liver stiffness measurement by transient elastography. Metabolic dysfunction-associated steatotic liver disease (MASLD) was diagnosed according to EASL guidelines. C-reactive protein (CRP) was measured as a marker of systemic inflammation. Multivariable linear regression and mediation analysis were performed, adjusting for age and sex. Results: Malnutrition was present in 50.6% of patients, MASLD in 66.7%, and both conditions coexisted in 36.8%. Malnutrition (B = 2.29, *p* < 0.001), MASLD (B = 1.54, *p* = 0.001), smoking (B = 1.06, *p* = 0.014), and CRP (B = 0.32, *p* < 0.001) were independently associated with increased liver stiffness. CRP partially mediated the effect of malnutrition on liver stiffness (indirect effect = 0.54; 95% CI 0.20–0.95), accounting for 18% of the total effect. Conclusions: In T2DM, malnutrition is a strong independent predictor of liver fibrosis, with its effect partially mediated by systemic inflammation. Addressing nutritional status and inflammatory burden may help slow fibrotic progression in this high-risk population.

## 1. Introduction

Malnutrition is common in type 2 diabetes mellitus (T2DM) and is associated with reduced quality of life, higher hospitalisation rates, disability, and increased all-cause and cardiovascular mortality [1,2,3,4]. In this population, poor nutritional status accelerates skeletal muscle loss, a key driver of sarcopenia [5]. Loss of muscle mass contributes to a chronic inflammatory state, which worsens metabolic dysfunction and insulin resistance, creating a self-perpetuating cycle [6,7].

In this scenario, chronic low-grade inflammation is a hallmark of T2DM and plays a major role in the progression of diabetic complications, particularly liver disease [8,9]. Inflammatory cytokines such as IL-6, TNF-α, and CRP are consistently elevated in T2DM and promote lipid accumulation, oxidative stress and fibrosis [10,11,12,13].

Metabolic dysfunction-associated steatotic liver disease (MASLD)—formerly known as NAFLD—is highly prevalent in T2DM, with fibrosis representing the main prognostic determinant of long-term liver-related outcomes [14,15]. Malnutrition and inflammation engage in a dynamic, bidirectional interaction that amplifies their respective effects: poor nutritional status exacerbates systemic inflammation, while persistent inflammation worsens malnutrition via cytokine-mediated mechanisms [16].

Emerging evidence suggests that malnutrition, like inflammation, may be directly implicated in the pathogenesis of liver fibrosis by impairing immune regulation and antioxidant defences [17].

Malnutrition could amplify both hepatic and systemic inflammatory responses, leading to hepatic stellate cell activation, extracellular matrix deposition, and fibrogenesis [18].

However, the relative contribution of malnutrition, inflammation, and their interaction to the development of liver fibrosis in T2DM remains poorly defined. To date, no studies have explored whether systemic inflammation mediates the relationship between malnutrition and liver stiffness in patients with T2DM. This study aimed to evaluate the association between malnutrition and liver fibrosis, and to determine whether systemic inflammation mediates this relationship.

## 2. Materials and Methods

### 2.1. Study Design

A prospective, single-centre study was conducted in the Liver Unit of University of Foggia, Foggia, Italy. We recorded clinical data from 87 adults consecutively attending outpatient clinics between January and June 2025 and affected by T2DM. Inclusion criteria were age ≥ 18 years and diagnosis of T2DM. Exclusion criteria included active malignancy, acute infection, pre-existing chronic liver diseases (including viral or autoimmune hepatitis), and cirrhosis. The study protocol was approved by the Institutional Review Board of Policlinico Riuniti (Foggia, Italy), and all procedures were conducted in accordance with the Declaration of Helsinki.

At enrolment, venous blood samples were obtained to measure haemoglobin, total white blood cell count (WBC), neutrophil count, lymphocyte count, platelet count, glucose, albumin, creatinine, HbA1c, total cholesterol, low-density lipoprotein (LDL), high-density lipoprotein (HDL), triglycerides, and C-reactive protein (CRP).

The triglyceride–glucose (TyG) index was calculated using $\ln\frac{fasting\;triglycerides\;[mg/dL]\;\times \;fasting\;glucose\;[mg/dL]\;}{2}$ [19].

Body mass index (BMI) was calculated as weight (kg) divided by height squared (m^2^).

Comorbidities assessed included hypertension, chronic kidney disease (CKD), heart failure, ischaemic chronic heart disease, atrial fibrillation, stroke, and chronic obstructive pulmonary disease (COPD), as well as self-reported smoking habits. Type 2 diabetes mellitus (T2DM) was diagnosed in the presence of at least one of the following criteria: fasting plasma glucose ≥ 126 mg/dL (7.0 mmol/L) confirmed on two separate occasions; glycated hemoglobin (HbA1c) ≥ 6.5%; or a 2-h plasma glucose value ≥ 200 mg/dL (11.1 mmol/L) during a standard 75 g oral glucose tolerance test (OGTT). Ongoing treatment with antidiabetic agents was also considered as evidence of diabetes; however, since metformin can be prescribed for other conditions such as polycystic ovary syndrome, obesity, or prediabetes, patients on metformin alone were classified as diabetic only if at least one of the above biochemical criteria was satisfied. Metabolic dysfunction-associated steatotic liver disease (MASLD) was defined according to EASL 2024 guidelines [20]. The Charlson Comorbidity Index was used to quantify comorbidity burden.

This study was performed in line with the principles of the Declaration of Helsinki. Approval was granted by the Ethics Committee of “Policlinico Riuniti” in Foggia (N◦161/CE/2023, approval date 23 October 2023). Informed consent was obtained from all participants in their medical records.

### 2.2. Nutritional Assessment

Nutritional status was evaluated using the Subjective Global Assessment (SGA), which incorporates both subjective and objective components. The SGA was chosen because it is validated across the full adult age range, including both adults under 65 and older adults, and it is widely used in outpatient populations with chronic conditions such as T2DM [21,22]. The subjective evaluation covered changes in dietary intake, unintentional weight loss, gastrointestinal symptoms affecting oral intake (e.g., diarrhoea, vomiting, nausea, dysphagia, oral issues), and functional capacity. The objective evaluation involved physical examination for loss of subcutaneous fat, muscle wasting, and presence of oedema or ascites. Patients were classified as A (well nourished), B (moderate malnutrition), or C (severe malnutrition) [21]. For analysis, categories B and C were combined into a single ‘malnutrition’ category.

### 2.3. Liver Stiffness and Steatosis Assessment

Liver stiffness was measured by transient elastography (FibroScan^®^ 630 Touch, Echosens, Paris, France) after ≥3 h of fasting. Only measurements with ≥10 valid acquisitions, a success rate > 60%, and an interquartile range/median ratio < 0.30 were considered reliable. Steatosis was assessed using the Controlled Attenuation Parameter (CAP). A cut-off of 248 dB/m was adopted, as this value corresponds to the optimal Youden-index threshold proposed by Karlas et al. for detecting ≥ S1 steatosis and has been widely validated across multiple cohorts. This cut-off was selected to ensure high sensitivity for identifying early steatotic changes, consistent with the aims of the present study [20,23].

### 2.4. Statistical Analysis

Continuous variables were tested for normality and expressed as mean ± standard deviation or median (interquartile range) as appropriate. Categorical variables were expressed as number (percentage). Group comparisons were performed using independent-samples t-test for normally distributed continuous variables, Mann–Whitney U test for non-normally distributed variables, and chi-square test for categorical variables.

A priori sample-size considerations were performed using G*Power vers 3.197 (linear multiple regression, fixed model, single regression coefficient). Assuming a medium effect size (f^2^ = 0.15), α = 0.05, two-tailed testing, power = 0.80, and four predictors of interest, the required sample size was estimated at 55 participants.

Multiple linear regression was used to examine associations between nutritional status, inflammatory markers, and metabolic parameters with liver stiffness. Variables with *p* < 0.05 in age- and sex-adjusted univariate models were entered into the multivariable model, which was further adjusted for age and sex.

**Figure 1:**
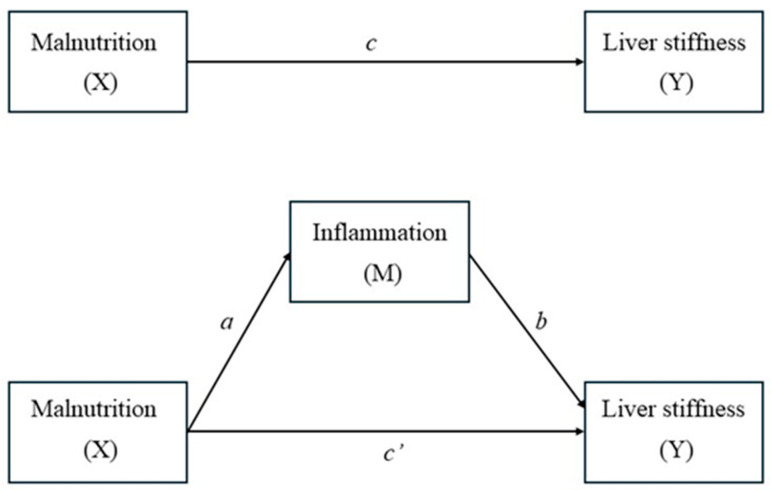
Conceptual framework for the mediation analysis. Path *c* (top panel) represents the total effect of malnutrition (X) on liver stiffness (Y). Paths *a* and *b* represent the indirect effect of malnutrition on liver stiffness via inflammation (M). Path *c*′ represents the direct effect of malnutrition on liver stiffness after adjusting for inflammation.

Mediation analysis was performed using the non-parametric bootstrap method (5000 resamples) to test whether CRP mediated the association between malnutrition and liver stiffness (Figure 1). The proportion mediated was calculated as the ratio of the indirect effect to the total effect. Statistical significance was set at two-sided *p* ≤ 0.05. Analyses were performed using R (version 4.3) within the RStudio 2025.09.2+418 environment (Posit Software, Boston, MA, USA).

## 3. Results

### 3.1. Demographic and Clinical Characteristics of the Study Population

**Figure 2:**
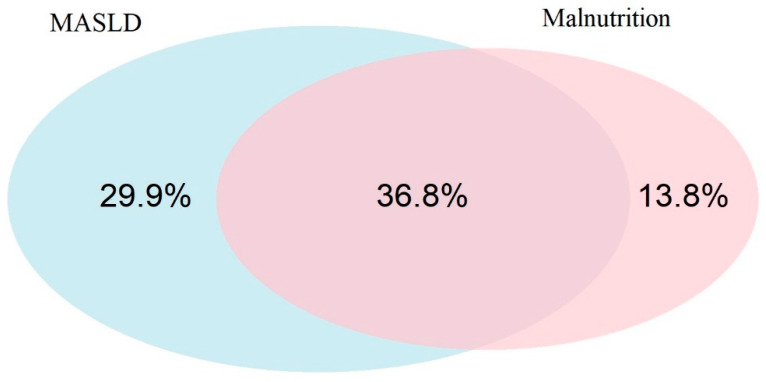
Venn diagram illustrates the distribution of malnutrition and MASLD in the study cohort (*n* = 87). A total of 32 patients (36.8%) presented with both MASLD and malnutrition. Twenty-six patients (29.9%) had MASLD without malnutrition, while 12 patients (13.8%) were malnourished without MASLD.

In this prospective observational study, a total of 87 patients were enrolled, of whom 28 (32.2%) were female. The median age was 67 years [IQR: 60–72]. According to SGA, 44 patients (50.6%) were classified as malnourished, while the remaining 49.4% were considered well-nourished. The overall prevalence of MASLD in the cohort was 66.7%. An analysis of the overlap between MASLD and malnutrition revealed that 36.8% of patients were affected by both conditions. Interestingly, 29.9% had MASLD in the absence of malnutrition, while 13.8% were malnourished without signs of MASLD. These findings are visually summarized in the Venn diagram (Figure 2).

The most frequent comorbidities were dyslipidaemia (80.5%) and hypertension (62.1%). Other conditions, including chronic kidney disease, ischemic heart disease, atrial fibrillation, COPD, and stroke, were less prevalent. Smoking was reported by 27.6% of patients. Data are summarized in Table 1.

Patients with malnutrition showed lower levels of WBC, neutrophils, lymphocytes, total cholesterol, and LDL cholesterol, as well as higher levels of HbA1c, CRP, and the TyG index. They also exhibited a greater comorbidity burden, as indicated by higher Charlson Comorbidity Index scores. Moreover, those with impaired nutritional status showed significantly higher liver stiffness values. Baseline characteristics are reported in Table 2.

### 3.2. Association of Nutritional and Inflammatory Factors with Liver Fibrosis

To explore potential predictors of liver fibrosis, we conducted a multiple linear regression analysis using liver stiffness as the dependent variable. Initially, several clinical and biochemical parameters were assessed through univariate analyses. Variables found to be statistically significant were subsequently included in the multivariable model (Table 3). All analyses were adjusted for age and sex to account for potential confounding.

**Figure 3:**
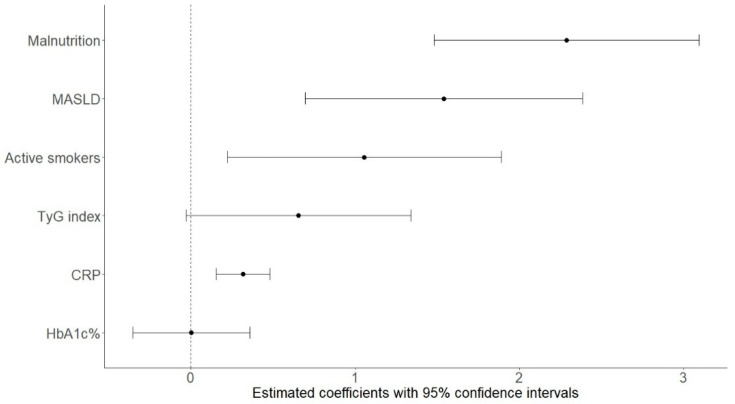
Forest plot showing the estimated coefficients and 95% confidence intervals from the multivariable linear regression model for predictors of liver stiffness in patients with T2DM. Abbreviations: MASLD, metabolic dysfunction-associated steatotic liver disease; TyG index, triglyceride–glucose index; CRP, C-reactive protein.

In the final model, malnutrition emerged as the strongest independent predictor of increased liver stiffness (B = 2.29, *p* < 0.001). This was followed by the presence of MASLD (B = 1.54, *p* = 0.001) and smoking status (B = 1.06, *p* = 0.014). Additionally, CRP was significantly associated with liver stiffness (B = 0.32, *p* < 0.001). In contrast, neither the TyG index nor HbA1c showed significant independent associations (Figure 3). A comprehensive summary of the regression results is provided in Table 3.

### 3.3. Mediation Analysis

We investigated whether inflammation, assessed by CRP levels, acted as a mediator in the relationship between malnutrition and liver stiffness, adjusting for age and sex.

The total effect of malnutrition on liver stiffness was statistically significant (B = 2.97, SE = 0.47, t = 6.35, *p* < 0.001), F(3, 83) = 13.78, *p* < 0.001, R^2^ = 0.33, indicating that poor nutritional status was associated with greater liver stiffness. When CRP was added to the model as a mediator, the association between malnutrition and liver stiffness remained significant (B = 2.42, SE = 0.46, *p* < 0.001) but was attenuated, suggesting partial mediation. This mediation model was significant, F(4, 82) = 15.41, *p* < 0.001, R^2^ = 0.43. Malnutrition was positively associated with CRP levels (B = 1.64, SE = 0.53, *p* = 0.003), F(3, 83) = 3.67, *p* = 0.015, R^2^ = 0.12, and CRP, in turn, was positively associated with liver stiffness (B = 0.33, SE = 0.09, *p* < 0.001), after adjusting for malnutrition and covariates.

**Figure 4:**
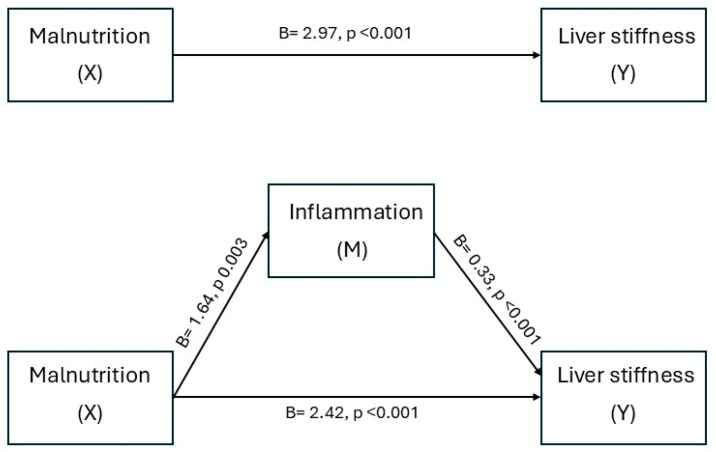
Mediation model of the association between malnutrition (X) and liver stiffness (Y) via inflammation (M). Unstandardized regression coefficients (B) and *p*-values are shown for each path. Solid arrows indicate statistically significant effects. All models adjusted for age and sex.

The indirect effect of malnutrition on liver stiffness via CRP was statistically significant (Effect = 0.54, BootSE = 0.20, 95% CI [0.20, 0.95]). The partially standardized indirect effect was 0.21 (95% CI [0.08, 0.37]). This indirect path accounted for approximately 18% of the total association between malnutrition and liver stiffness. Figure 4 illustrates the path diagram with unstandardized coefficients and *p*-values for each link.

**Figure 5:**
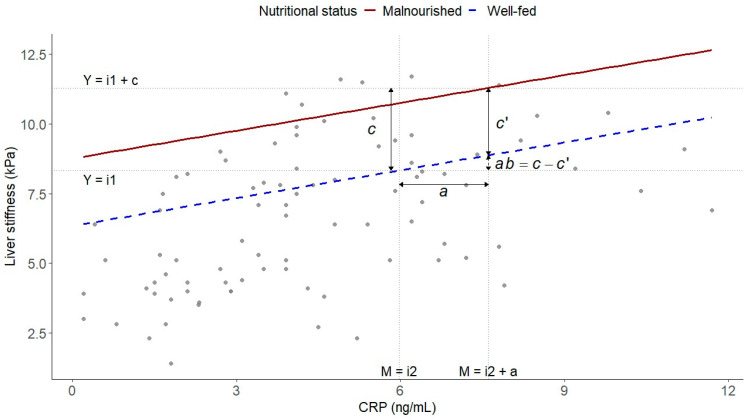
Graphical representation of the mediation mechanism.

Following the framework proposed by Fritz and MacKinnon [24], Figure 5 depicts liver stiffness (Y-axis) against CRP levels (X-axis), with separate regression lines for participants with low (dashed blue) and high (solid red) malnutrition scores. The horizontal distance between vertical reference lines corresponds to the “*a*” path (effect of malnutrition on CRP), while the slope of each regression line represents the “*b*” path (effect of CRP on liver stiffness, controlling for malnutrition). The vertical gap between regression lines at a given CRP value reflects the direct effect of malnutrition (*c*′), whereas the total effect (*c*) represents the association without accounting for CRP. The difference between *c* and *c*′ corresponds to the mediated (indirect) component of the association.

The figure displays liver stiffness (Y-axis) as a function of CRP levels (X-axis), with separate regression lines for individuals with high (red solid line) and low (blue dashed line) malnutrition. The slopes reflect the association between CRP and liver stiffness within each malnutrition group.

## 4. Discussion

In this prospective study of patients with T2DM, we found that malnutrition was strongly and independently associated with higher liver stiffness, even after accounting for age, sex, MASLD, and other comorbidities. Importantly, systemic inflammation, as reflected by CRP levels, partially mediated this relationship, suggesting a mechanistic link between poor nutritional status and progression of hepatic fibrosis in this high-risk population.

Our findings add to a growing body of evidence indicating that malnutrition is not only common in individuals with T2DM, but also clinically relevant for liver-related outcomes [4,25].

In our cohort, malnourished patients showed higher CRP levels and lower lymphocyte counts, consistent with the effect of malnutrition on inflammatory status. The significantly lower total and LDL cholesterol observed in malnourished patients is coherent with the poor nutritional status. These biochemical differences further highlight the close interplay between malnutrition and systemic inflammation in T2DM.

Current evidence indicates that malnourished patients with chronic liver disease have a more rapid progression of fibrosis, increased rates of complications, and reduced survival compared with well-nourished patients [26]. By evaluating an outpatient diabetic population without advanced liver disease, we found that the effects of malnutrition on liver stiffness are already detectable, with higher mean values compared with well-nourished counterparts. This suggests that malnutrition may exert a measurable hepatic impact before progression to advanced stages. The partial mediation by CRP observed in our analysis supports a mechanistic pathway whereby malnutrition may contribute to fibrosis progression through heightened systemic inflammation. These findings address an important evidence gap and support the concept that malnutrition should be considered a relevant risk factor for hepatic fibrosis even in community-dwelling patients with T2DM. Malnutrition may exacerbate systemic inflammation through impaired immune function, reduced antioxidant capacity, and increased susceptibility to infections [27,28,29].

Conversely, chronic low-grade inflammation can worsen nutritional status via cytokine-induced anorexia, increased resting energy expenditure, and muscle catabolism [30,31]. This reciprocal relationship creates a self-perpetuating cycle that may also involve the liver, where heightened systemic inflammation contributes to hepatic stellate cell activation, extracellular matrix deposition, and accelerated fibrosis progression [32].

From a pathophysiological perspective, the role of inflammation in fibrogenesis is well established. IL-6, TNF-α, and CRP have been implicated in the activation of hepatic stellate cells, modulation of Kupffer cell activity, and alteration of the hepatic microenvironment [33,34]. In addition, inflammation has been shown to promote hepatic steatosis through cytokine-mediated metabolic alterations and oxidative stress, and steatosis itself is a recognized risk factor for fibrosis progression [35,36]. In the setting of T2DM and MASLD, persistent metabolic stress and oxidative injury may synergize with these inflammatory pathways, amplifying fibrosis progression [37]. In our cohort, the persistence of an independent association between CRP and liver stiffness—even after adjusting for MASLD—suggests that inflammatory pathways exert fibrogenic effects beyond those attributable to metabolic steatosis alone. An additional finding of our multivariable analysis was that active smoking was independently associated with increased liver stiffness, regardless of malnutrition, MASLD, and systemic inflammation. This aligns with prior evidence linking smoking to oxidative stress, immune dysregulation, and hepatic microvascular alterations, all of which may drive hepatic stellate cell activation and extracellular matrix deposition [38,39,40]. Smoking contributes to adverse lipid and inflammatory alterations closely linked to insulin resistance, providing an additional plausible pathway through which smoking may exacerbate liver injury in T2DM [41]. In T2DM, smoking can also amplify chronic low-grade inflammation and worsen endothelial dysfunction, thereby accelerating fibrogenesis [42].

Clinically, these findings underscore the need for integrated management strategies in T2DM that address both nutritional status and inflammatory burden. A proactive approach should include early screening for malnutrition, personalised dietary counselling, and targeted nutritional interventions to preserve lean body mass, optimise protein quality and quantity, and correct micronutrient deficiencies. Nutritional interventions aimed at preserving lean body mass, improving protein intake, and correcting micronutrient deficiencies could potentially mitigate systemic inflammation and slow fibrosis progression [43,44,45]. Anti-inflammatory dietary patterns, such as the Mediterranean diet, have been shown to reduce CRP levels and improve metabolic parameters in T2DM [46]. Improving micronutrient status may also help mitigate systemic inflammation and improve redox balance in T2DM [47]. In addition, previous evidence showed that low levels of vitamin D are associated with increased pro-inflammatory cytokine expression and greater liver fibrosis in patients with chronic liver disease [48]. Future studies should specifically investigate if serum vitamin D levels in malnourished patients with diabetes correlate with liver stiffness progression. Our results also have implications for MASLD risk stratification. While fibrosis remains the strongest predictor of liver-related outcomes, recent data suggest that higher degrees of steatosis may be associated with increased mortality and cardiometabolic complications [49,50].

Thus, a combined evaluation of steatosis burden, fibrosis stage, and nutritional status may provide a more comprehensive risk profile for patients with T2DM.

This study has some limitations. First, the sample size and drawn from a single care centre, which may limit generalizability. Second, CRP is a nonspecific marker of inflammation; future studies incorporating a broader panel of inflammatory cytokines may provide a more detailed mechanistic understanding. Third, the duration of antidiabetic therapy was not considered, which could potentially influence the degree of liver fibrosis. Lastly, the cross-sectional nature of liver stiffness assessment precludes definitive conclusions about causality, although the biological plausibility of the proposed pathway is supported by experimental and clinical evidence. Moreover, we adopted 248 dB/m as CAP threshold for steatosis detection, privileging sensitivity for ≥S1. This cut-off was originally proposed by Karlas et al. [23] as the optimal Youden index value for detecting any degree of steatosis, and has since been validated in several cohorts as a reliable threshold to rule out S0. Given our study aim to capture the earliest hepatic changes associated with malnutrition in T2DM, maximizing sensitivity for even mild steatosis was prioritized. We acknowledge that higher cut-offs (≈275–280 dB/m) are often used to ‘rule in’ moderate-to-severe steatosis with higher PPV, and this methodological choice may affect comparability with other cohorts.

## 5. Conclusions

Malnutrition is a strong and independent predictor of liver stiffness in patients with T2DM, and this association is partially mediated by systemic inflammation. Early identification and correction of nutritional deficits, coupled with strategies to reduce chronic inflammation, may represent a novel and cost-effective approach to attenuate fibrosis progression and improve long-term outcomes in this vulnerable population. Our findings also support incorporating early malnutrition screening and simple inflammatory markers into the routine MASLD work-up for patients with T2DM, as these elements may enhance clinical risk stratification and guide more timely preventive interventions. Large, longitudinal studies are needed to confirm these findings and determine whether early nutritional and anti-inflammatory interventions can translate into improved liver-related and overall outcomes

## Figures and Tables

**Table 1:** Prevalence of comorbidities in the study population.

Comorbidities	*n*
Hypertension	54 (62)
Dyslipidemia	70 (80.5)
Chronic Kidney Disease	11 (12.6)
Ischemic Heart Disease	8 (9.2)
Atrial fibrillation	6 (6.9)
Heart failure	6 (6.9)
Chronic obstructive pulmonary disease	4 (4.6)
Stroke	3 (3.4)
Active smokers	24 (27.6%)

Data are represented as *n* (%).

**Table 2:** Baseline characteristics of the patients according to nutritional status.

	Well-Fed (SGA A) n. 43 (49.4%)	Malnourished (SGA B + C) n. 44 (50.6%)	*p* Value
Age, years	66.0 [55.0–71.0]	68.0 [62.0–74.0]	0.118
Genre F, *n* (%)	15 (34.9)	13 (29.5)	0.651
BMI, Kg/m^2^	27.7 ± 5.6	29.2 ± 6.1	0.227
Haemoglobin, g/dL	14.4 ± 1.8	13.6 ± 2.2	0.066
WBC, *n*/mm^3^	7389 ± 2802	6148 ± 2053	**0.022**
Neutrophils, *n*/mm^3^	4518 ± 1909	3751 ± 1463	**0.038**
Lymphocytes, *n*/mm^3^	2060 [1570–2540]	1660 [1100–2275]	**0.041**
Platelet, 10^3^/mcL	234 ± 72	202 ± 88	0.072
Glucose, mg/dL	126.8 ± 25.4	130.1 ± 38.3	0.065
Albumin, g/dL	4.4 ± 0.5	4.3 ± 0.5	0.132
Creatinine, mg/dL	0.94 ± 0.33	1.02 ± 0.5	0.384
HbA1c, %	7.1 ± 1.0	7.8 ± 1.21	**0.010**
Total cholesterol, mg/dL	174.9 ± 38.0	144.9 ± 35.7	**<0.001**
LDL, mg/dL	105.3 ± 28.7	78.1 ± 27.4	**<0.001**
HDL, mg/dL	47.3 ± 10.4	57.4 ± 14.4	0.977
Triglycerides, mg/dL	91 [79–130]	92 [65–138]	0.975
CRP, ng/mL	3.5 [2.1–5.4]	4.4 [3.1–6.7]	**0.018**
TyG index	8.5 ± 0.5	8.8 ± 0.7	**0.050**
Charlson index	5 [3–7]	7 [5–9]	**0.042**
Active smokers	13 (30.2)	11 (25%)	0.637
MASLD	26 (60.5)	32 (72.7)	0.261
SGLT2-i/GLP-1 agonist	14 (32.6)	23 (52.3)	0.083
Liver stiffness, kPa	5.3 ± 2.2	8.0 ± 2.1	**<0.001**
CAP, dB/m	251.8 ± 61.8	271.5 ± 60.6	0.336

Data are reported as mean (±SD), median [IQR] or *n* (%) as appropriate. Abbreviations: F, female; WBC, white blood cells; LDL, low-density lipoprotein; HDL, high-density lipoprotein; CAP (Controlled attenuation parameter). *p* value < 0.05 were considered statistically significant (in bold).

**Table 3:** Linear regression analyses evaluating the impact of nutritional status, inflammation, and metabolic risk on liver stiffness.

Univariate Linear Regression				Multivariate Linear Regression		
	Coefficient B	95% CI	*p* Value	Coefficient B	95% CI	*p* Value
BMI, Kg/m^2^	0.07	−0.03–0.17	0.179			
HbA1c, %	0.59	0.120–1.05	**0.014**	0.01	−0.36–0.36	0.993
CRP, ng/mL	0.48	0.29–0.98	**<0.001**	0.32	0.15–0.48	**<0.001**
TyG index	1.65	0.79–2.50	**<0.001**	0.65	−0.03–1.34	0.061
Hypertension	0.86	−0.28–1.99	0.138			
CKD	−0.75	−2.47–0.98	0.392			
Dyslipidemia	0.24	−1.17–1.64	0.737			
Heart failure	0.49	−1.79–2.78	0.669			
ICHD	−0.96	−2.91–0.99	0.329			
Atrial fibrillation	0.44	−1.79–2.68	0.695			
Stroke	0.53	−2.54–3.60	0.732			
COPD	−1.28	−3.96–1.39	0.343			
MASLD	2.30	1.21–3.39	**<0.001**	1.54	0.70–2.38	**0.001**
Malnutrition	2.97	2.04–3.90	**<0.001**	2.29	1.48–3.10	**<0.001**
Charlson index	0.07	−0.12–0.26	0.449			
Active smokers	1.24	0.02–2.46	0.047	1.06	0.22–1.89	**0.014**
SGLT2-i/GLP-1 agonist	1.1	−0.03–2.24	0.06			

Univariate and multivariate models were performed to explore associations with liver stiffness. Abbreviations: CRP, C-reactive protein; CKD, chronic kidney disease; ICHD, ischemic heart disease; COPD, chronic obstructive pulmonary disease; MASLD, metabolic dysfunction-associated steatotic liver disease; TyG, triglyceride–glucose index; HbA1c, glycated hemoglobin; SGLT2-i, sodium-glucose cotransporter-2 inhibitor; GLP-1, glucagon-like peptide-1 receptor agonist.

## Data Availability

The dataset analysed during the current study is available from the corresponding author on reasonable request due privacy restrictions.
